# Investigation of the efficacy and feasibility of combined therapy of PD‐L1‐enhanced exogenous peripatetic adoptive natural killer (NK) cells in combination with antiangiogenic targeted therapy in the treatment of extensive‐stage small cell lung cancer

**DOI:** 10.1111/1759-7714.15040

**Published:** 2023-08-19

**Authors:** Zhizhen Wang, Ruiping Zhang, Yuchan Cao, Yang Chen, Sheng Huang, Yan'an Luo

**Affiliations:** ^1^ Tianjin Medical University Cancer Institute & Hospital, National Clinical Research Center for Cancer, Tianjin's Clinical Research Center for Cancer Tianjin China; ^2^ Brown University School of Public Health Brown University Providence Rhode Island USA; ^3^ Nankai University Tianjin China

**Keywords:** antiangiogenesis, extensive‐stage small‐cell lung cancer, immune checkpoint inhibitor, PD‐1/PD‐L1, radiotherapy

## Abstract

A 67‐year‐old male patient presented with extensive‐stage small cell lung cancer with the primary lesion located in the right upper lung, accompanied by multiple metastases to the pleura and abdominal cavity with enlarged mediastinal lymph nodes. A combination therapy approach was used to target the patient's multiple systemic metastases after localized radiotherapy. The approach involved adoptive transfer of programmed death ligand 1 (PD‐L1) enhanced exogenous natural killer (NK) cells, along with antiangiogenic treatment. Allogeneic cord blood NK cells were infused back into the patient over two consecutive days. On the first day, the treatment was followed by a dose of 1200 mg of atezolizumab. Subsequently, the patient received a daily dose of 10 mg of anlotinib administered orally for 14 days. This was followed by a 7‐day break, and each cycle lasted 21 days. After delivering localized radiation to the primary lesion in the right lung and metastatic mediastinal lymph nodes, complete remission was achieved in the local lesion, effectively avoiding the risk of superior vena cava syndrome. Following six cycles of combined therapy, most of the metastatic lesions had disappeared, and the remaining metastatic lesions had significantly reduced in size. The recent therapeutic effect resulted in partial remission. The combination therapy of immune checkpoint inhibitor PD‐L1‐enhanced exogenous adoptive transfer NK cells, along with antiangiogenic targeted treatment, demonstrated a satisfactory short‐term effect, with disappearance of most of the metastases and noticeable shrinkage in the remaining metastatic lesions.

## Graphical abstract

In this study, we investigated the efficacy and feasibility of combined therapy of PD‐L1 enhanced exogenous peripatetic adoptive NK cells in combination with anti‐angiogenic targeted therapy in the treatment of extensive‐stage small‐cell lung cancer. The patient reported in the article can not tolerated systemic chemotherapy, and subsequently chose to immunosuppressive agent PS‐L1 enhanced exogenous adoptive NK cells combined with anti‐angiogenic targeted drugs, and undergone locally radiotherapy for high‐risk local lesions. The recent clinical observation showed significant efficacy in practice.

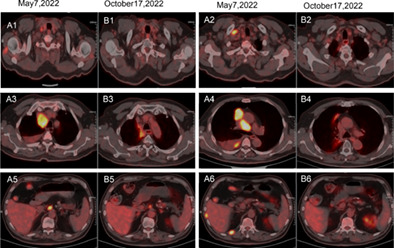

## INTRODUCTION

Lung cancer still remains the most common solid tumor in China, with ~482 000 new cases and 321 000 deaths in 2022.^1^ Small cell lung cancer (SCLC) accounts for 15%–20% of all lung cancer cases, and around 70% of SCLC patients are diagnosed with distant metastases.^2^, ^3^ SCLC is classified into two stages based on the extent of cancer cell spread: limited‐stage small cell lung cancer (LS‐SCLC) and extensive‐stage small cell lung cancer (ES‐SCLC). While SCLC is highly responsive to radiotherapy and chemotherapy, achieving short‐term remission is common. However, it is prone to local recurrence and distant metastasis. LS‐SCLC can be treated with local radiotherapy and systemic chemotherapy, but determining the optimal treatment for ES‐SCLC is particularly challenging. Currently, systemic chemotherapy remains the primary treatment for ES‐SCLC due to its extensive spread and limited efficacy of local radiotherapy. However, the outcomes of this approach are unsatisfactory, with a published 5‐year survival rate of less than 1%.^2^

In recent years, there has been a glimmer of hope in the field of immunotherapy for the treatment of solid tumors. The combination of immunotherapy with chemotherapy or the use of single immunotherapy agents has emerged as a new treatment option for patients with ES‐SCLC. However, there is still a need to explore and develop more optimized treatment modalities. Unfortunately, the task presents a challenge for clinical physicians due to the limited availability of real‐world clinical experience in this area. Furthermore, even with the current options, the treatment outcomes using immunotherapy are still not satisfactory. There is a pressing need for further research and advancements in order to improve the effectiveness of immunotherapy in ES‐SCLC.

Here, we report a case study of a patient with ES‐SCLC who was unable to tolerate systemic chemotherapy. As a result, a treatment approach involving the use of an immunosuppressive agent called programmed death ligand 1 (PD‐L1)‐enhanced exogenous adoptive natural killer (NK) cells was chosen, along with antiangiogenic targeted drugs. Additionally, local radiotherapy was administered to target high‐risk local lesions. The recent clinical observation has demonstrated significant efficacy in the patient's case.

The primary objectives of the report were to conduct a preliminary exploration of the multidimensional selection of systemic treatment options for ES‐SCLC and to investigate the value of immunotherapy in this context. Additionally, the second aim was to identify the most effective combination treatment agent when used in conjunction with immunotherapy.

## CASE REPORT

**FIGURE 1:**
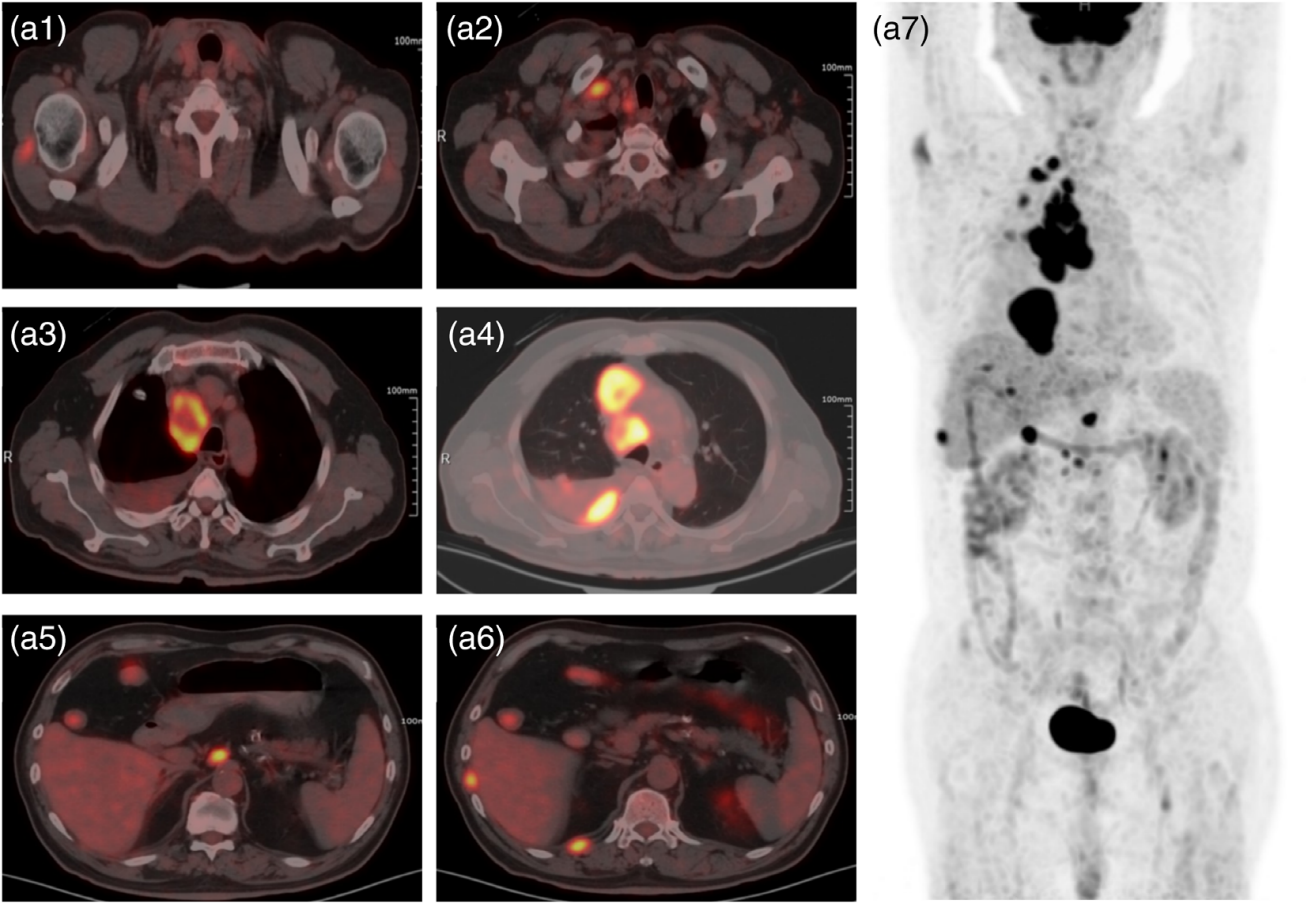
Pretreatment ^18^F‐fluorodeoxyglucose positron emission tomography/computed tomography (^18^F‐FDG PET/CT) images taken in May 2022. (a1) A slightly hypodense nodal shadow in the right shoulder deltoid, which does not exclude metastasis. (a2) Lymph nodes in the right clavicular area. (a3, a4) Intramediastinal and paratracheal lymph nodes with right pleural effusion. (a5) Parietal abdominal aorta and perivascular lymph nodes in the inferior abdominal aorta and inferior vena cava. (a6) Nodules under the right anterior wall and 11–12th intercostal space and multiple small nodular mass‐like thickening of the right pleura, considered to be pleural metastasis. (a7) The patient's whole body ^18^F‐FDG metabolic map.

A 67‐year‐old Chinese male patient underwent a routine physical examination in September 2021, during which a computed tomography (CT) scan revealed the presence of a nodule located beneath the pleura of the right upper lobe of the lung. No immediate treatment was given at that time. In January 2022, the patient began experiencing symptoms including hoarseness, coughing, and shortness of breath. The symptoms improved after self‐administration of anti‐inflammatory treatment. However, by May 2022, the patient's coughing and shortness of breath had worsened. An ^18^F‐fluorodeoxyglucose positron emission tomography/computed tomography (^18^F‐FDG PET/CT) scan revealed a high metabolic rate in a nodule located in the right upper lobe of the lung, strongly suggesting a high probability of lung cancer, as shown in Figure 1. The scanning showed increase metabolic activity in multiple lymph nodes located in various regions, including the right supraclavicular area, mediastinum, trachea, para‐aortic region, and inferior vena cava. Furthermore, the image also revealed the presence of multiple nodular‐thickening lesions in the right pleura, which exhibited increased metabolic activity. The findings suggested pleural metastasis, accompanied by right pleural effusion. Additionally, local nodular thickening lesions with increased metabolic activity were observed in the peritoneum, indicating peritoneal metastasis. A slightly lower‐density nodular lesion with increased metabolic activity was also identified in the right shoulder triangle, and while metastasis could not be definitively ruled out, it remained a possibility.

In 2018, the patient experienced severe liver function damage as a result of consuming Chinese herbal medicine wine. While the patient's condition was improved after receiving professional treatment, it led to bone marrow suppression and significant reductions in platelets and white blood cell counts. Additionally, the patient had a 25‐year history of hypertension, with the highest recorded blood pressure reaching 160/100 mmHg. However, the hypertension was well‐controlled through appropriate management. In 2011, the patient was diagnosed with coronary heart disease and subsequently underwent a stent implantation procedure in the left anterior descending artery. Then, in 2017, the patient underwent a cholecystectomy to address gallbladder disease and made a good recovery. Following the current admission, a biopsy of the right clavicular lymph node was conducted, and based on the results of pathological examination, imaging studies, and immunohistochemistry, the patient was diagnosed with metastatic small cell lung cancer. The immunohistochemistry results were as follows: CKpan(+), TTF(+), CgA(slightly+), Syn(−), CD56(+), Ki67 (about 90%), and P40(−). The report on the tumor markers prior to treatment is presented in Table 1.

**TABLE 1:** Changes in tumor biomarkers before and during treatment.

Date	Gastrin‐releasing peptide precursor (ng/L)	Neuron‐specific enolase (ug/L)	Carcinoembryonic antigen (ug/L)	Cytokeratin 19 fragment (ug/L)
May 10, 2022	2263.26	62.1	41	3.48
June 7, 2022	160.72	17.0	17.8	2.53
August 10, 2022	110.63	11.9	6.52	4.19
October 17, 2022	196.12	18.3	7.54	3.99

*Note*: The first row represents the biomarker data before the start of radiotherapy. The second row represents the data after 20 fractions of radiotherapy. The third and fourth rows represent the biomarker data during follow‐up.

**FIGURE 2:**
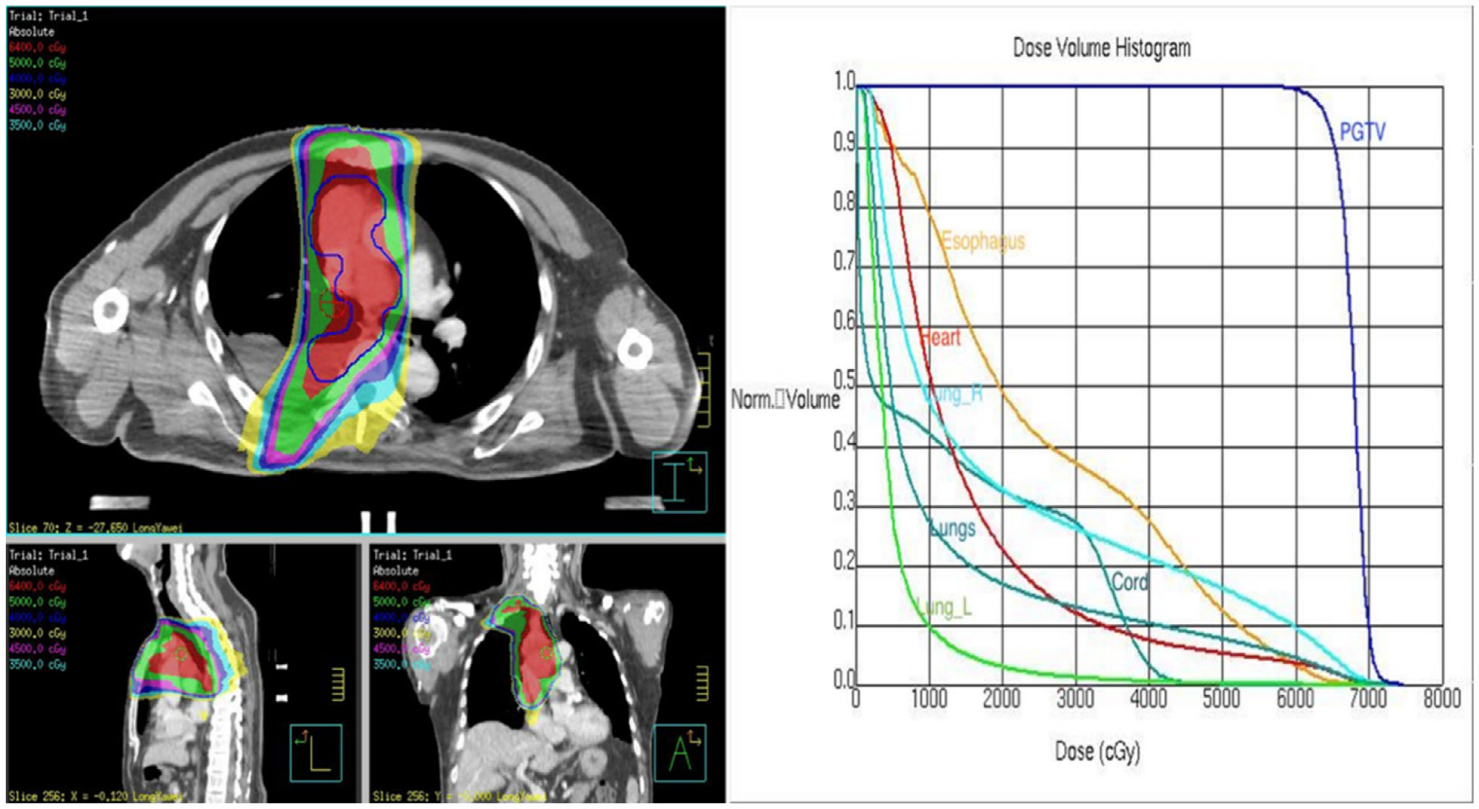
Dose distribution and dose‐volume histogram (DVH) of the patient's radiotherapy plan. In the left image, the dose distribution is showed in transverse, sagittal and frontal planes, respectively. The dose delivered to target named planning gross tumor volume (PGTV) and organ at risk (OAR), including esophagus, heart, lung_R, lung_L, and cord can be seen on the DVH.

The patient's local lung lesion was accompanied by mediastinal lymph node metastasis, especially in close proximity to the superior vena cava. To prevent the development of superior vena cava syndrome, the patient underwent radiotherapy targeting the right lung lesion, mediastinum, and lymph nodes on the right side. The prescribed dose was 60 Gy, administered in a total of 30 fractions with five fractions delivered each week. The dose distribution and dose‐volume histogram (DVH) can be observed in Figure 2.

**FIGURE 3:**
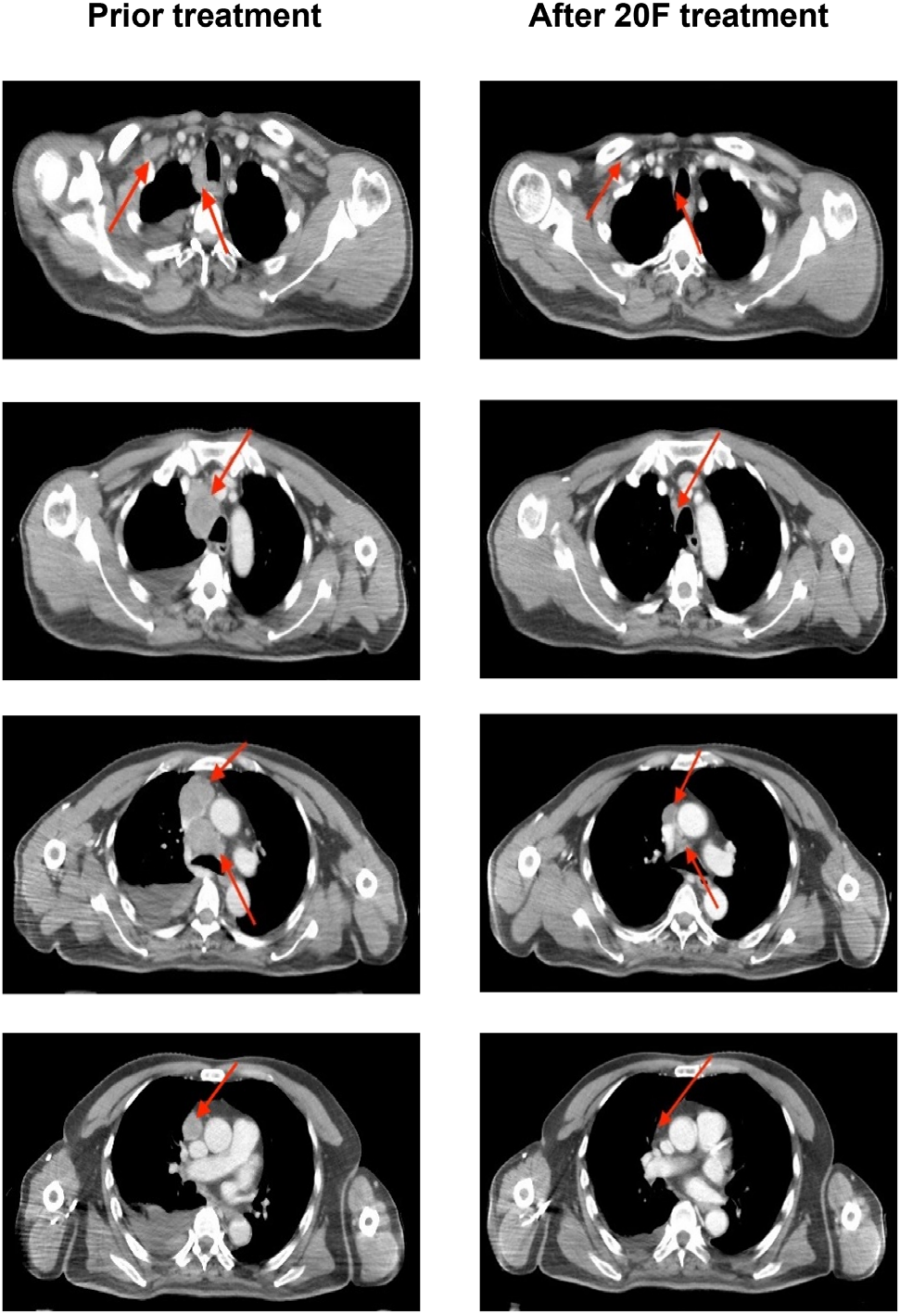
Computed tomography (CT) images of the patient before radiotherapy and after 20 fractions of radiotherapy. The first column displays the CT images taken prior to radiotherapy, while the follow‐up CT images after 20 fractions (F) of radiotherapy are shown in the second column. All images are presented using a window width of 400 and a window level of 800. The tumor area is indicated by red arrows in both the pretreatment and post 20F treatment images.

Taking into consideration the patient's history of serious liver function abnormalities and second‐degree bone marrow suppression, along with low platelet count (below 60 × 10^9^/L) and low white blood cell count (below 3 × 10^9^/L), the use of chemotherapy posed an increased risk of liver damage and further bone marrow suppression. Therefore, a combination of immunotherapy using atezolizumab (Genentech, Inc.) and targeted therapy with anlotinib (Nanjing Chia Tai Tianqing Pharmaceutical Co., Ltd.) was chosen as the treatment approach. Atezolizumab was administered at a dose of 1200 mg on the first day only, followed by a treatment break. Anlotinib, on the other hand, was taken orally at a dose of 10 mg daily for 14 consecutive days, followed by a 7‐day break, constituting a treatment cycle of 21 days. After receiving 20 fractions of radiotherapy, a significant reduction in the size of the right lung lesion, as well as the mediastinum and lymph nodes on the right side, was observed in the CT image shown in Figure 3. The decrease in the tumor marker after 20 fractions of radiotherapy is displayed in Table 1.

The patient's symptoms improved significantly after undergoing radiotherapy. At this point, exogenous adoptive NK cell therapy was introduced as part of the systemic treatment plan. The specific protocol involved culturing allogeneic cord blood NK cells for ~2 weeks. Subsequently, these NK cells were combined with atezolizumab at a dosage of 1200 mg. The cultured NK cells, totaling 3–5 billion, were divided into two infusions without any pretreatment prior to administration. On the first day, the patient received NK cells along with atezolizumab, while on the second day, only NK cells were infused into the patient's body. This cycle was repeated every 21 days. In addition, the patient continued to receive an oral dose of 10 mg anlotinib daily for 14 days, followed by a 7‐day break, constituting a treatment cycle of 21 days.

A follow‐up CT scan conducted on August 10, 2022, compared to the previous ^18^F‐FDG PET/CT scan conducted on May 7, 2022, revealed several positive changes. The right pleural effusion had decreased, the thickening of the right pleura had reduced, and the size of mediastinal and right supraclavicular lymph nodes had also decreased. Furthermore, no significant enlargement of lymph nodes in the abdomen or retroperitoneum was observed. The follow‐up tumor marker is shown in Table 1.

**FIGURE 4:**
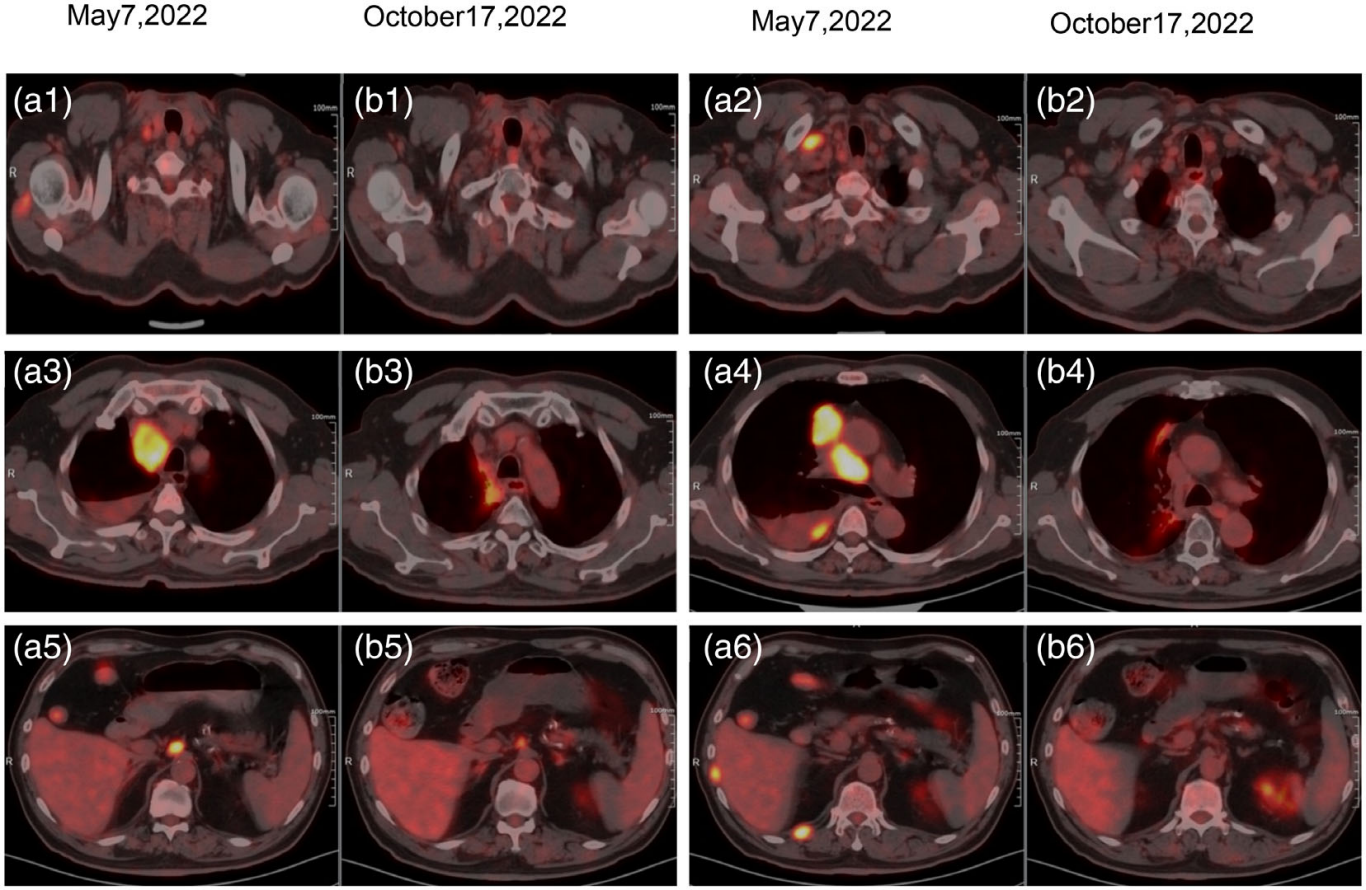
Comparison of ^18^F‐fluorodeoxyglucose positron emission tomography/computed tomography (^18^F‐FDG PET‐CT) images after 5 months following PD‐L1‐enhanced exogenous pericytes combined with antiangiogenic targeting therapy. (a1–a6) The patient's pretreatment images from May 7, 2022. (b1–b6) The patient's images from October 17, 2022.

In comparison to the previous scan conducted on May 7, 2022, the subsequent ^18^F‐FDG PET/CT scan performed on October 17, 2022, demonstrated notable volume reduction. Specifically, shrinkage was observed in the nodule located in the right upper lobe, as well as in the multiple lymph nodes situated in the right supraclavicular and mediastinal region near the trachea, vessels, para‐aortic artery, and inferior vena cava. These changes are shown in Figure 4. Despite the positive response observed on the previous scan, it is worth noting that two lymph nodes adjacent to the para‐aortic artery exhibited persistent increased metabolic activity on the most recent ^18^F‐FDG PET/CT scan. However, there was a decrease in the thickening of multiple small nodes on the right pleura, accompanied by a reduction in both metabolic activity and the presence of right pleural effusion.

Furthermore, there was a significant decrease in the previously observed nodular thickening in the local peritoneum, right anterior chest wall, and the 11th–12th rib space. Additionally, the slightly low‐density nodular shadows in the right shoulder muscle also showed a substantial reduction. These findings indicated that the systemic treatment had been effective, resulting in the disappearance of most of the metastases and noticeable shrinkage in the remaining metastatic lesions. Imaging assessments suggested that the therapeutic regimen had been successful, and the tumor was in a stage of partial remission. The results of the tumor marker test are presented in Table 1.

## DISCUSSION

There are often challenges in the treatment of patients with ES‐SCLC due to the large tumor burden from widespread metastases and the limited effectiveness of local treatments. Systemic drug therapy, primarily chemotherapy, is the mainstay of treatment for these patients. However, the prognosis remains concerning even after chemotherapy. Based on the clinical outcomes observed in the case reported here, the short‐term combination of immunosuppressive PD‐L1‐enhanced exogenous NK cells and antiangiogenic targeted therapy demonstrated promising results. This approach led to the disappearance of most of the metastases and significant shrinkage of the remaining metastatic lesions. These findings suggest that combination therapy can achieve a positive effect in the short term, which offers new hope for the treatment of ES‐SCLC patients in the future.

### ES‐SCLC chemoradiotherapy

Etoposide and cisplatin (EP) or etoposide and carboplatin (EC) are the established standard first‐line chemotherapy regimens for SCLC, as recommended by the National Comprehensive Cancer Network (NCCN).^3^ However, in the case reported in this study, the patient had a history of severe liver damage and bone marrow suppression, which rendered them unable to tolerate traditional chemotherapy after a clinical evaluation. As a result, conventional systemic treatment options were not suitable for this particular case. Therefore, it is crucial to explore alternative effective systemic treatments for patient with such specific conditions and limitations.

For SCLC patients, local radiotherapy has demonstrated a high control rate and can even lead to curative outcomes. When compared to chemotherapy alone, the combination of chemotherapy and radiotherapy has been shown to significantly improve overall survival (OS). Furthermore, synchronous chemoradiotherapy has been found to result in longer OS compared to sequential chemoradiotherapy.^4^, ^5^ However, for ES‐SCLC patients, the primary treatment approach revolves around systemic drug therapy, with radiotherapy being utilized selectively to address specific symptoms or to prevent the development of serious symptoms. The role of radiotherapy in ES‐SCLC is primarily limited to provide the necessary assistance in managing symptoms and is not intended to achieve comprehensive therapeutic effectiveness against the disease itself. In the case described in the study, the patient initially presented with extensive metastatic lesions in the neck, chest, and abdomen, including a significant volume of mediastinal lymph nodes near the vena cava. In order to prevent the occurrence of serious symptoms associated with superior vena cava syndrome, local radiotherapy was administered. Subsequently, systemic drug therapy was employed to control the widespread metastasis throughout the body.

### ES‐SCLC immunotherapy

Immunotherapy has emerged as a significant breakthrough in cancer treatment. Historically, for SCLC, there have been limited advancements in recent years. However, one of the most promising developments has been the use of immune checkpoint inhibitors, which target the mechanisms that allow tumors to evade the immune system. Programmed cell death protein 1 (PD‐1) is an important immune checkpoint receptor that, when activated, suppresses T cell activity. PD‐L1 is the corresponding ligand for PD‐1. Antibodies targeting the PD‐L1/PD‐1 pathway have demonstrated therapeutic efficacy in various cancer through immune checkpoint blockade. These agents have been extensively studied in clinical trials and have gained widespread recognition in clinical practice. In addition to PD‐L1/PD‐1 immune checkpoint inhibitors, combination therapies targeting multiple pathways and utilizing multiple targets have shown potential for achieving improved treatment outcomes. It is important to explore various treatment strategies to optimize the therapeutic response. The results of the phase III checkmate227 trial^6^ demonstrated that dual immunotherapy with nivolumab and ipilimumab in advanced EGFR/ALK‐negative non‐small cell lung cancer (NSCLC) patients led to better OS compared to traditional chemotherapy regimens. However, it is worth noting that the checkmate032^7^ and checkmate451^8^ studies did not show a significant survival benefit and had increased toxic reactions when dual immunotherapy was used for SCLC. These findings highlight the complexity of immunotherapy and the need for further research to identify the most effective treatment approaches for different cancer types.

### PD‐L1/PD‐1 combined with chemotherapy

The results in the studies conducted by Bilani et al.^9^ and Peters et al.^10^ demonstrated that the use of immunosuppressive drugs (nivolumab and ipilimumab) did not result in improved progression‐free survival (PFS) for LS‐SCLC following clinical therapy. These findings indicate that monotherapy of immunotherapy has not been shown to enhance the prognosis of SCLC.

Atezolizumab is an immune checkpoint inhibitor that targets PD‐L1. The IMpowerl33^11^, ^12^ study is the first global multicenter randomized double‐blind phase III to demonstrate an improvement in OS in first‐line treatment of ES‐SCLC using atezolizumab. Subsequent updates of the study have further confirmed these results.^13^ As a result, the NCCN guidelines recommend atezolizumab in combination with EC as a first‐line treatment for ES‐SCLC. The combination of chemotherapy and immunotherapy has brought new hope. Other studies^14^, ^15^ have also conducted a global multicenter randomized open‐label phase III clinical trial for the first‐line treatment of ES‐SCLC, and have confirmed the effectiveness of combining chemotherapy and immunotherapy.

The results of several clinical trials, such as the keynote‐604 phase III study^16^ with pembrolizumab, the phase III checkmate 331 study with nivolumab,^17^ the phase II IFCT‐1603 study with atezolizumab,^18^ and the phase II keynote‐158 study with keytruda,^19^ have shown negative outcomes for single‐agent immunotherapy or combination chemotherapy in the treatment of ES‐SCLC.

Based on the current evidence, single‐agent immunotherapy has not demonstrated significant clinical benefit for SCLC patients, and combination chemotherapy has shown only limited efficacy. Finding more effective combination therapies for ES‐SCLC remains a challenging task. It is worth noting that for patients who cannot tolerate chemotherapy, immunotherapy alone may not be effective, as evidenced by the case reported in this article. Therefore, exploring alternative treatment strategies for ES‐SCLC, especially for patients intolerant to chemotherapy, is essential to improve outcomes for this challenging disease.

### PD‐L1/PD‐1 immune checkpoint inhibitors combination with antiangiogenic target

Antiangiogenic therapy is an important treatment option for advanced lung cancer. Vascular endothelial growth factor (VEGF) and its receptor (VEGFR) play crucial roles in this process tumor by promoting the formation of abnormal tumor blood vessels, leading to increased microvascular permeability, enhanced endothelial cell proliferation, and augmented endothelial cell migration.^20^ Several drugs, such as anlotinib, apatinib, aegorafenib, and bevacizumab, have demonstrated significant efficacy in various solid tumors, including those treated with target‐based immunotherapy. Anlotinib, for instance, prolonged PFS to 4.1 months compared to only 0.7 months with placebo in the study by Cheng et al.^21^, ^22^ Moreover, anlotinib reduced the risk of disease progression by 81% without significantly increasing related adverse events. Additionally, patients in the anlotinib group had a median OS of 7 months, whereas the placebo group had a median OS of 4 months. Based on these findings, the CSCO Primary Lung Cancer Diagnosis and Treatment Guidelines recommend anlotinib as a second‐line or higher treatment option for SCLC. Anlotinib has been recently approved as a new drug for SCLC in China and may be considered a preferred option for combination with targeted and immunotherapeutic approaches.

A phase II single‐arm clinical study conducted in Shanghai investigated the combination of anlotinib with either etoposide or cisplatin for ES‐SCLC.^23^ The study aimed to explore the potential benefits of this combination therapy. The results demonstrated encouraging outcomes, with an overall response rate (ORR) of 90% and disease control rate (DCR) of 96.7%. The median PFS was 6 months, and median OS was 14 months. Similar clinical observations were also conducted, yielding consistent findings. Another study reported an ORR of 90%, a DCR of 100%, a median PFS of 10.3 months, and a median OS of 17.1 months.^24^ These results further confirm the efficacy and safety of combining anlotinib with chemotherapy.

Preliminary evidence from small‐scale clinical observations suggests that combining anlotinib with immunotherapy has demonstrated initial effectiveness in the treatment of SCLC.^25^, ^26^ Currently, phase II clinical trials are underway to further evaluate the combination of anlotinib with PD‐1 inhibitors (NCT04055792), toripalimab (NCT04620837), and treprostinil (NCT04731909) for SCLC, including both advanced and extensive‐stage cases.

In the case reported here, the patient was unable to tolerate concurrent chemotherapy. Fortunately, there is promising evidence supporting the combination of anlotinib with immunotherapy, and anlotinib is recommended as a second‐line treatment option for SCLC. Therefore, the treatment approach for this case involved combining immunotherapy with anlotinib to provide a potential therapeutic benefit.

### PD‐L1/PD‐1 combination with adoptive NK cells

PD‐L1/PD‐1 immune checkpoint inhibitors have addressed the issue of immune recognition in tumor cells. When there is only one abnormality in the antigen recognition step of the tumor immune cycle, monotherapy with PD‐L1/PD‐1 immune checkpoint inhibitors can achieve good efficacy. However, in the tumor immune cycle, multiple steps may have different degrees of abnormalities, which collectively contribute to the tumor growth. It is speculated that combining multiple treatment modalities may address the abnormalities in different steps of the tumor immune cycles and lead to improved efficacy.

Combination therapy, particularly the supplementation of normal functional immune T cells to improve both the quantity and function of T cells, has become the best choice for SCLC patients. The combination of PD‐L1/PD‐1 immune checkpoint inhibition with adoptive cellular therapy based on NK cells is theoretically feasible and may offer potential benefit.

A study found that the infusion of allogeneic donor NK cells in 15 patients with NSCLC led to partial tumor shrinkage in two patients and stable disease in six patients.^27^ In NSCLC patients with nivolumab treatment, higher absolute numbers of circulating NK cells were associated with longer OS.^28^ Several human studies have demonstrated that PD‐1 expression on NK cells is present in cancer patients and weakens their antitumor effects. However, the use of immune checkpoint inhibitors can enhance the effect of NK cells.^29^

PD‐1/PD‐L1 strongly suppresses the antitumor immune activity mediated by NK cells, and PD‐1‐expressing and NK cell infiltrated tumors often exhibit functional exhaustion. Studies have shown that introducing PD‐1 blocking antibodies into NK cells enhances therapeutic efficacy. In an in vitro activity study using a mouse model of multiple myeloma, the effectiveness of PD‐1/PD‐L1 inhibitor in reversing NK cell exhaustion and improving their function was confirmed.^30^

In animal studies, the combination of anti‐PD‐L1 and PM21‐NK cells resulted in significant improved OS compared to treatment with anti‐PD‐L1 alone (48 vs. 24 days, *p* = 0.0001).^31^ A clinical study conducted in China reported on the combination of the first anti‐PD‐1 antibody (pembrolizumab) with allogeneic NK cell therapy in advanced NSCLC patients.^32^ The results showed that combination therapy led to a doubling of NK cell numbers and a significant decrease in cytokineslevels (IL‐2, TNF‐β, and IFN‐γ) and circulating tumor cells (CTCs), which are tumor markers. The overall response rate in the combination therapy group was significantly higher (36.5% vs. 18.5%), and the survival outcomes were also significantly better than those of the anti‐PD‐1 antibody alone group (OS: 15.5 vs. 13.3 months; PFS: 6.5 vs. 4.3 months; both of *p* < 0.05). Moreover, the benefit of the combination therapy was more pronounced in patients with a tumor proportion score (TPS) ≥ 50%. Patients receiving multiple courses of NK cell infusion showed better OS than those with a single course of NK cell infusion (18.5 vs. 13.5 months). Therefore, the combination of anti‐PD‐L1 antibodies and NK cells can significantly enhance antitumor activity and improve survival outcomes. PD‐1/PD‐L1 antibodies can help alleviate the functional impairments of NK cells caused by the tumor microenvironment, restore their cytotoxic activity against tumors, and even enhance T cell‐mediated antitumor immunity. NK cells can also indirectly enhance the efficacy of PD‐1/PD‐L1 blockade by affecting other immune cells in the tumor microenvironment.^33^ Hence, this strategy may be beneficial in improving the effectiveness of immune therapy.

Ongoing phase I/II clinical trials^34^ are investigating the combination of NK cells with PD‐1 antibodies (sintilimab and pembrolizumab) in patients with advanced NSCLC (NCT03958097) and advanced cholangiocarcinoma (NCT03937895). NK cells can be obtained from various sources, such as NK‐92, KHYG‐1, NKL, and NKG.^35^ They constitute ~10% of peripheral blood lymphocytes and around 30% in umbilical cord blood (UCB), making UCB a rich source of NK cells for adoptive therapy.^36^

Previous trials combing NK cell with PD‐1 blockade have shown promising clinical prospects. Both autologous and allogeneic NK cells can address the immune cell function deficiencies. However, the source cells for culture expansion may have functional impairments that are not fully resolved. It can be inferred that the therapeutic effect of allogeneic NK cell infusion may be better than that of autologous infusion, but further trials are needed to verify this.

A combination therapy approach involving antiangiogenesis (anlotinib) and PD‐L1/PD‐1 immune suppression (atezolizumab) along with adoptive cell immunotherapy was attempted in the case reported in this study. The results showed partial remission after three cycles of treatment, especially in widely metastatic lesions, demonstrating excellent efficacy that surpassed traditional standard treatment. To our knowledge, no significant treatment efficacy or similar treatment plans have been found for ES‐SCLC without chemotherapy. The probability of achieving a curative effect using a single drug is limited in theory, and combination therapy may hold the key to success in this case. However, it is necessary to expand the number of cases and conduct prospective randomized controlled observations for further validation in the future.

Here, we present the first reported case of a combination treatment approach that considers the immune circulation system at multiple stages, surpassing other combined treatment modalities involving PD‐1/PD‐L1 immune inhibitors besides chemotherapy. This approach should be considered for patients with ES‐SCLC. It presents a potentially effective supplementary treatment option beyond chemotherapy.

## AUTHOR CONTRIBUTIONS

Zhizhen Wang and Ruiping Zhang and Yuchan Cao wrote and edited the draft of manuscript. Yang Chen and Yuchan Cao collected all the images. Sheng Huang and Yan'an Luo revised the draft critically. Ruiping Zhang provided the idea and revised the case report finally. All authors read and approved the final manuscript.

## CONFLICT OF INTEREST STATEMENT

The authors declare that they have no competing interests.
